# Experimental validation of the RATE tool for inferring HLA restrictions of T cell epitopes

**DOI:** 10.1186/s12865-017-0204-1

**Published:** 2017-06-21

**Authors:** Sinu Paul, Cecilia S. Lindestam Arlehamn, Veronique Schulten, Luise Westernberg, John Sidney, Bjoern Peters, Alessandro Sette

**Affiliations:** Division of Vaccine Discovery, La Jolla Institute for Allergy and Immunology, 9420 Athena Circle, La Jolla, San Diego, CA 92037 USA

**Keywords:** HLA restriction, HLA association, MHC, Epitope, T cell, RATE

## Abstract

**Background:**

The RATE tool was recently developed to computationally infer the HLA restriction of given epitopes from immune response data of HLA typed subjects without additional cumbersome experimentation.

**Results:**

Here, RATE was validated using experimentally defined restriction data from a set of 191 tuberculosis-derived epitopes and 63 healthy individuals with MTB infection from the Western Cape Region of South Africa. Using this experimental dataset, the parameters utilized by the RATE tool to infer restriction were optimized, which included relative frequency (RF) of the subjects responding to a given epitope and expressing a given allele as compared to the general test population and the associated *p*-value in a Fisher’s exact test. We also examined the potential for further optimization based on the predicted binding affinity of epitopes to potential restricting HLA alleles, and the absolute number of individuals expressing a given allele and responding to the specific epitope. Different statistical measures, including Matthew’s correlation coefficient, accuracy, sensitivity and specificity were used to evaluate performance of RATE as a function of these criteria. Based on our results we recommend selection of HLA restrictions with cutoffs of *p*-value < 0.01 and RF ≥ 1.3. The usefulness of the tool was demonstrated by inferring new HLA restrictions for epitope sets where restrictions could not be experimentally determined due to lack of necessary cell lines and for an additional data set related to recognition of pollen derived epitopes from allergic patients.

**Conclusions:**

Experimental data sets were used to validate RATE tool and the parameters used by the RATE tool to infer restriction were optimized. New HLA restrictions were identified using the optimized RATE tool.

**Electronic supplementary material:**

The online version of this article (doi:10.1186/s12865-017-0204-1) contains supplementary material, which is available to authorized users.

## Background

Identification of HLA alleles restricting specific T cell epitopes is an important component of accurate characterization of T cell responses. This information is required, for example, for the production of tetramer staining reagents [1–3], or to evaluate association of particular HLAs with protective or predisposing T cell responses [4–6]. The restricting HLA alleles can be determined by experiments relying on classical immunological approaches, such as inhibition by HLA locus specific antibodies, and use of matched/mismatched or single HLA allele transfected cell lines [7]. These experimental approaches can be time consuming and resource intensive. As an alternative, we developed a computational method called RATE (Restrictor Analysis Tool for Epitopes) that infers HLA restriction of epitopes from T cell response data in HLA typed subjects [8]. “T cell response data” is the specific immune response to various epitopes in PBMCs from HLA typed individuals measured by, for example, IFN-γ ELISPOT and reported as spot-forming cells (SFCs) per million cells. RATE infers HLA restrictions by considering the presence or absence of a response to a given epitope as the biological outcome, and calculating the relative frequency (RF) of the subjects responding to a given epitope and expressing a given allele as compared to the general test population and associated statistical significance.

This method was initially validated with a small set of experimental data, generated to verify a limited number of inferred restrictions, and by retrospective analysis of data sets publicly available online. We recently reported the results of a clinical study for which HLA restrictions were experimentally determined for 191 *Mycobacterium tuberculosis* (MTB) peptides tested in a South African cohort of 63 MTB infected individuals by the use of single HLA transfectants [9]. This provided an opportunity for an unbiased validation of RATE, and also for further optimization of its performance by systematically examining the effect of varying different parameters linked to the analysis and its output. The subsequently updated version of the RATE tool server has also been made available online (http://iedb-rate.liai.org/).

## Materials and methods

### Study subjects and peptides

The study involved MTB-specific T cell response data from healthy adults with latent MTB infection from the Worcester region of the Western Cape Province of South Africa, as detailed in Arlehamn et al. (2016) [9]. The responses studied are resulting from natural exposure to whole TB. MTB donors were recruited based on IGRA (Interferon gamma release assay; FDA approved for diagnosis of latent TB infection) reactivity and lack of active TB symptoms. Donors with a positive IGRA are latently infected with MTB. Peptides representing the vaccine candidate and IGRA antigens (Rv3874; CFP10 and Rv3875; ESAT-6) (15-mers overlapping by 10 amino acids spanning each entire protein) and epitopes from the frequently recognized antigens previously reported by Arlehamn et al. [10], as well as additional frequently recognized epitopes described in ex vivo experiments and available in the IEDB database (www.iedb.org) [11–15] were included in the study as described previously [9].

Data on allergen-epitope T cell reactivity independently and previously reported from a separate cohort of Timothy grass (TG) allergic donors from the Denver, CO and San Diego, CA regions was also investigated. Donors had a skin prick test of > 3 mm to Timothy grass or a TG-specific IgE titer of > 0.35 kU/L and a clinical history of seasonal allergic symptoms consistent with Timothy grass pollen allergy [16–19]. Immunodominant peptides from Timothy grass pollen T cell antigens were studied, as well as peptides from other grasses, including Kentucky blue grass, Rye grass, Canary grass and Orchard grass. These peptides were conserved in grass pollen across species and elicited responses in two or more pollen allergic patients [16–19]. Peptides were synthesized as crude material on a small (1 mg) scale by A and A (San Diego).

### PBMC isolation, ELISPOT assays and HLA typing

Peripheral blood mononuclear cells (PBMC) were purified from whole blood by layering onto Ficoll and density-gradient centrifugation, according to the manufacturer’s instructions.

Cells were cryopreserved in liquid nitrogen suspended in FBS containing 10% (vol/vol) DMSO.

For ELISPOT assays, PBMC were stimulated at 2 × 10^5^ cells/well in triplicate with peptide pools (5 μg/ml), peptides (10 μg/ml), PHA (10 μg/ml) or medium containing 0.25% DMSO (percent DMSO in the pools, as a control) in 96-well plates (Immobilion-P; Millipore) coated with 5 μg/ml anti-IFNγ (1-D1K; Mabtech). After 20 h incubation at 37 °C, wells were washed with PBS/0.05% Tween 20 and incubated with biotinylated anti-IFNγ (7-B6-1; Mabtech) for 2 h. Spots were developed using Vectastain APC peroxidase (Vector Laboratories) and 3-amino-9-ethylcarbazole (Sigma-Aldrich). Spots were counted by computer-assisted image analysis (KS-ELISPOT reader; Zeiss). Responses were considered positive if the net spot-forming cells (SFC) per 10^6^ PBMC were ≥20, the stimulation index ≥2, and *p* ≤0.05 (Student’s *t*-test, mean of triplicate values of the response against relevant pools or peptides vs. the DMSO control). All samples had a viability >75%, as determined by trypan blue, and reactivity to PHA >400 SFC/10^6^ cells.

Four-digit HLA typing for these cohorts was done as previously described [20]. Genomic DNA was isolated from PBMC using standard techniques (REPLI-g; Qiagen). Amplicons for HLA class I and class II genes were generated using PCR and locus-specific primers. Amplicons of the correct size were purified using Zymo DNA Clean-up Kit, according to the manufacturer’s instructions. Sequencing libraries were prepared using Nextera XT reagents (Illumina), according to manufacturer’s instructions. The libraries were purified using AMPure XP (Beckman Coulter) with a ratio of 0.5:1 beads to DNA (vol/vol). The libraries were pooled in equimolar amounts and loaded at 5.4pM on one MiSeq flowcell with 1% phiX spiked in (MiSeq Reagent Kit v3). Paired-end sequencing was performed with 300 cycles in each direction. HLA typing calls were made using HLATyphon (https://github.com/LJI-Bioinformatics/HLATyphon).

### HLA restriction using single HLA transfected cell lines

HLA restriction assays using single HLA transfected cell lines were performed as described earlier [9]. Single HLA transfected RM3 (derived from human B lymphocyte cell line Raji) or DAP.3 (L cell fibroblast) were maintained in culture. In preparation for the assay, the cell lines were harvested and viability (all >75%) was determined using Trypan Blue. Each cell line at 2x10^5^ cells/well was pulsed with 10 μg/ml individual peptide for 1 h at 37 °C, followed by four washes in RPMI. PBMC at 2x10^5^/well were stimulated in triplicate with peptide pulsed cell line (5x10^4^ cells/well), cell line alone (as a control), peptides (10 μg/ml), PHA (10 μg/ml) or medium containing 0.25% DMSO (percent DMSO in the peptides, as a control) in 96-well plates (Immobilion-P; Millipore) coated with anti-IFNγ antibody as described above for single cytokine ELISPOT. Criteria for positive responses were as described for ELISPOT assays above.

### RATE calculations

The RATE tool (http://iedb-rate.liai.org/) [8] was used to computationally infer the HLA restrictions from the immune response and HLA typing data described above. RATE estimates Relative Frequency (RF) to quantify the strength of associations between expression of a specific allele and detection of positive immune response. An RF > 1 indicates a positive association between the two properties in question (i.e., expressing the specific allele increases the “odds” of having positive immune response). RF is calculated according to the formula:

$$ R F=\frac{A^{+}{R}^{+}/\left({A}^{+}{R}^{+}+{A}^{+}{R}^{-}\right)}{\left({A}^{+}{R}^{+} + {A}^{-}{R}^{+}\right)/ Total\  donors} $$

Where

*A* ^*+*^ *R* ^*+*^ = Number of subjects who expressed a specific allele and gave a positive immune response to the specific peptide

*A* ^*-*^ *R* ^*-*^ = Number of subjects who did not express the specific allele and did not give a positive immune response to the specific peptide

*A* ^*-*^ *R* ^*+*^ = Number of subjects who did not express the specific allele but gave a positive immune response to the specific peptide

*A* ^*+*^ *R* ^*-*^ = Number of subjects who expressed the specific allele but did not give a positive immune response to the specific peptide

The Fisher’s exact test is used to estimate the statistical significance of the association between HLA molecules and epitope responses.

### Statistical evaluation of RATE results

In order to evaluate the performance of the RATE tool, the following statistical measures were estimated:

- Matthew’s Correlation Coefficient
  $$ M C C=\frac{\left( TP\times TN\right)-\left( FP\times FN\right)}{\sqrt{\left( TP+ FP\right)\left( TP+ FN\right)\left( TN+ FP\right)\left( TN+ FN\right)}} $$
  Where
*TP* = True positives
*FP* = False positives
*FN* = False negatives
*TN* = True negatives
- Accuracy
  $$ Accuracy=\frac{TP + TN}{Total} $$
- Sensitivity
  $$ Sensitivity=\frac{TP}{TP+ FN} $$
- Specificity
  $$ Specificity=\frac{TN}{FP+ TN} $$
- Precision
  $$ Precision=\frac{TP}{TP+ FP} $$
- False positive rate
  $$ F P R=\frac{FP}{FP+ TN} $$

## Results

### Assembly of an experimental data set for validation of the RATE tool

**Fig. 1:**
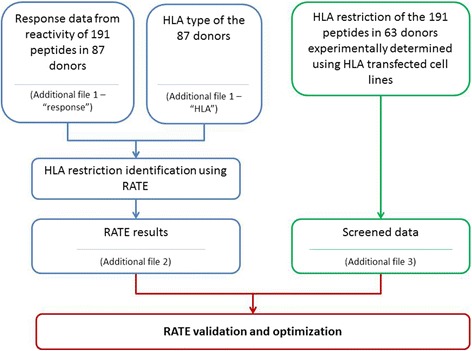
Illustrative description of the two sets of ELISPOT data derived from MTB epitopes and how they were used. The left panel shows the response data obtained when reactivity of each of the 191 peptides was determined in a set of 87 HLA typed donors and was used to infer restrictions by the RATE approach. The right panel shows the HLA restriction of the same 191 peptides in 63 donors determined experimentally using HLA transfected cell lines. This data was then screened and used for validation of RATE by comparing with RATE results from the first data set

The study involved two different sets of ELISPOT data derived from MTB epitopes (Fig. 1). The first set encompassed response data obtained when reactivity of each of the 191 peptides was determined in a set of 87 HLA typed donors for a total of 191 × 87 = 16,617 determinations (Additional file 1, tab “response”). This data, along with the HLA types of the 87 donors (Additional file 1, tab “HLA”) was utilized to infer restrictions by the RATE approach. The 87 donors expressed 111 unique HLA alleles and thus the RATE generated reports for 191 × 111 = 21,201 peptide/allele combinations (Additional file 2). The second data set entailed the experimental determination of HLA restriction of the same 191 peptides in 63 donors by the use of HLA transfected cell lines (7). Obviously, only peptides giving a positive response in a particular donor could be assessed for HLA restriction. Besides, not all possible combinations could be tested because HLA transfected cell lines were not available for some less frequent allelic variants. The HLA restriction was thus assayed for a total of 3,195 peptide/allele/donor combinations. Details on number of peptides, subjects and alleles are given in Table 1. To generate a robust data set for validating and optimizing RATE performance, only peptide/allele restrictions that were independently verified by positive experimental results in at least three experiments in different subjects were included as positive in the analysis. Likewise peptide/allele combinations consistently testing negative in multiple subjects were considered negative (non-restricting). The remaining peptide/allele combinations were considered as ambiguous and excluded from the validation analysis. This final data set contained 102 unique peptide/allele combinations (Additional file 3).

**Table 1:** Data sets used in the study. The table shows the number of peptides, subjects and alleles in each data set used in the study. The first set (column 1) encompassed response data, when reactivity of each of the 191 peptides was determined in a set of 87 HLA typed donors and was used to infer restrictions by the RATE approach. The second data set (column 2) entailed the experimental determination of HLA restriction of the same 191 peptides in 63 donors by the use of HLA transfected cell lines and was used in validation of RATE. The third data set (column 3) came from data on allergen-epitope T cell reactivity from a cohort of Timothy grass allergic donors

	ELISPOT data (for determining HLA restriction using RATE) (MTB)^a^	ELISPOT data (experimentally determined HLA restrictions)(MTB)^b^	Validation data (TG)
No. of peptides	191	191	66
No. of subjects/donors	87	63	137
No. of unique alleles	111	89	99
No. of allele-peptide combinations	21,201	16,999	6,534

^a^Additional file 1 shows the input data for RATE that was used for determining HLA restriction using RATE approach. Additional file 2 shows the complete results of this RATE analysis for all 21,201 peptide/allele combinations

^b^Based on availability of HLA transfected cell lines, restrictions were experimentally determined for 3,195 HLA/peptide/donor combinations. After screening of the data as mentioned in results, 102 entries were selected for validation. This data is shown in Additional file 3

### Experimental validation of the RATE tool

We next utilized this unbiased experimental data set to validate RATE. The RATE tool utilizes immune response data (in this case ELISPOT assay results) and HLA types of the subjects in which the various epitopes were tested to generate a list of parameters evaluating all possible HLA restrictions in terms of combinations of peptides and HLA alleles expressed by responding study subjects. These parameters include (1) Relative Frequency (RF) which is ratio of the response in subjects expressing the specific allele to the response in all donors (see Methods) (2) *p*-value indicating the statistical significance of RF in Fisher’s exact test and (3) *A* ^*+*^ *R* ^*+*^, defined as the number of subjects expressing the specific allele and having a positive response against a specific peptide.

The validation of RATE’s HLA restriction inference was done by comparing the RATE results generated from the MTB response data (Additional file 2) with the experimentally identified HLA restrictions (Additional file 3). The number of True Positives (TP), False Positives (FP), False Negatives (FN) and True Negatives (TN) were used to determine Matthew’s correlation coefficient (MCC), accuracy, sensitivity, specificity, precision, and false positive rate, as described in the Methods section. Since the data sets for validation are associated with binary outcomes (yes/no in terms of restriction and yes/no in terms of RATE predictions), MCC is more appropriate here than AUC values that are commonly used for statistical evaluation of predictive performance.

MCC can range from -1 to 1. -1 indicates perfect negative correlation, 0 random distributions and 1 perfect correlation. In general, MCC values of +0.70 or higher indicate a very strong positive relationship, +0.40 to +0.69 a strong positive relationship, +0.30 to +0.39 a moderate positive relationship, and values of +0.20 to +0.29 a weak positive relationship. Initially when all peptide/allele combinations with statistically significant RF (Fisher’s exact test *p*-value < 0.05) were selected as positive restrictions from RATE results, the Matthew’s correlation coefficient (MCC) was found to be 0.395 and had an accuracy of 0.706. The sensitivity, specificity and precision were 0.675, 0.726 and 0.614 respectively (Table 2). The False positive rate was 0.274. This indicated a moderately positive relationship between the allele restrictions provided by RATE tool and that identified experimentally.

**Table 2:** Effect of different cutoff values for *p*-value on RATE results. A cutoff of *p* <0.01 gave the best results with MCC = 0.451

Cutoffs	TP	FP	FN	TN	Total	Accuracy	Sensitivity	Specificity	Precision	False Positive Rate	Matthews Correlation Coefficient
*p* <0.05	27	17	13	45	102	0.706	0.675	0.726	0.614	0.274	0.395
*p* <0.01	22	8	18	54	102	0.745	0.550	0.871	0.733	0.129	0.451
*p* <0.005	18	5	22	57	102	0.735	0.450	0.919	0.783	0.081	0.432

### Optimization of RATE output as a function of *p*-value, RF and A^+^R^+^

We next used the experimental data set to identify the optimal cutoff values for *p*-value, RF and A^+^R^+^ parameters. As a first approach, the results were examined with cutoff for *p*-value varying between 0.05, 0.01 and 0.005 with no cutoffs being applied for other variables. The best MCC was obtained at *p* <0.01 (MCC = 0.451) (Table 2) and more stringent cutoffs were associated with lower overall performance and MCC values. The accuracy, specificity, and precision were 0.745, 0.871 and 0.733, respectively, while sensitivity, as a result of considering fewer potential restrictions as significant, was 0.550. The false positive rate stood at 0.129.

The RATE results were next examined for the effect of varying RF values. Since only RF ≥ 1.0 are associated with positive associations, it is reasonable to assume that a cutoff of RF ≥ 1.0 would be associated with higher performance. Selection of an overly high RF cutoff would, however, lead to significant reductions in TP-values and MCC. When the cutoff value for RF was varied between 1.0 and 2.5 with no other cutoffs being applied for other parameters, the best MCC was obtained at RF ≥ 1.3 (MCC = 0.314) (Table 3). For this cutoff, the accuracy, sensitivity, specificity, precision and false positive rate were 0.618, 0.825, 0.484, 0.508 and 0.516 respectively.

**Table 3:** Effect of different cutoff values for RF on RATE results. A cutoff of RF ≥ 1.3 gave the best results with MCC = 0.314

Cutoffs	TP	FP	FN	TN	Total	Accuracy	Sensitivity	Specificity	Precision	False Positive Rate	Matthews Correlation Coefficient
RF ≥ 1.00	34	44	6	18	102	0.510	0.850	0.290	0.436	0.710	0.162
RF ≥ 1.20	33	36	7	26	102	0.578	0.825	0.419	0.478	0.581	0.255
RF ≥ 1.25	33	35	7	27	102	0.588	0.825	0.435	0.485	0.565	0.270
RF ≥ 1.30	33	32	7	30	102	0.618	0.825	0.484	0.508	0.516	0.314
RF ≥ 1.35	31	31	9	31	102	0.608	0.775	0.500	0.500	0.500	0.275
RF ≥ 1.40	31	30	9	32	102	0.618	0.775	0.516	0.508	0.484	0.290
RF ≥ 1.45	30	29	10	33	102	0.618	0.750	0.532	0.508	0.468	0.279
RF ≥ 1.50	29	27	11	35	102	0.627	0.725	0.565	0.518	0.435	0.284
RF ≥ 1.75	26	24	14	38	102	0.627	0.650	0.613	0.520	0.387	0.257
RF ≥ 2.00	21	22	19	40	102	0.598	0.525	0.645	0.488	0.355	0.168
RF ≥ 2.50	16	15	24	47	102	0.618	0.400	0.758	0.516	0.242	0.168

We then examined the RATE performance as a function of A^+^R^+^ values ranging from 1 to 10 with no cutoffs being applied to other parameters. The MCC was found to be best at A^+^R^+^ ≥ 5. The MCC was 0.509 and the accuracy, sensitivity, specificity, precision and false positive rate were 0.725, 0.900, 0.613, 0.600 and 0.387 respectively (Additional file 4). This result suggests that focusing on HLA/peptide combinations with larger number of positive results inherently increases performance. However it should be noted that this parameter threshold has less practical utility, since the optimal performance will be different in data sets of different sizes (studies with different number of subjects being tested).

We next examined if different combinations of cutoffs for different parameters would improve the performance. The effect of different *p*-value cutoffs in combination with RF cutoffs was examined as an “OR” condition; namely considering restrictions positive if either a certain *p*-value or a certain RF value is met. The best MCC obtained was 0.314, when cutoffs RF ≥ 1.3 or *p*-value cutoffs 0.05, 0.01 and 0.005 were applied. Next, we considered combined cutoffs using an “AND” condition. When MCC was estimated with combination of *p*-value cutoffs 0.05, 0.01 and 0.005 with RF in the range of 1.0 to 2.0, it was found that the MCC of the combined cutoffs remained highest for *p* <0.01 in combination with RF values in the range of 1.2 to 1.75, with an MCC of 0.451. While this is not an improvement over the use of the *p*-value <0.01 by itself, we chose the combined cutoff of *p* <0.01 and RF ≥1.3 in order to have a more conservative and robust threshold. The cutoff for RF was chosen as ≥ 1.3 since this gave the best MCC when RF cutoffs were analyzed independently.

### Combination of RATE with HLA binding predictions does not yield further performance gains

We hypothesized that combining RATE outputs with HLA binding predictions, would improve the overall performance of RATE. To test this hypothesis, the effect of predicted HLA binding was investigated with the binding cutoff varied between IEDB consensus percentile ranks 5.0 and 25.0 without applying any other cutoffs (Additional file 5) and with cutoffs *p* <0.01 and RF ≥ 1.3. The MHC binding affinity for each peptide/allele combinations were predicted using IEDB MHC binding prediction tool (Kim et al., 2012). The lower numerical value of IEDB consensus percentile rank indicates stronger binding. Surprisingly, the incorporation of a predicted binding cutoff did not improve performance. The best MCC value was obtained for a consensus percentile rank of 15.0 when binding cutoffs were used without applying any other cutoffs and corresponded to an MCC value of 0.378 (Additional file 5). When predicted binding cutoffs were combined with cutoffs *p* <0.01 and RF ≥ 1.3, the MCC was actually lower, as compared to the MCC of 0.451 observed with *p* <0.01 and RF ≥ 1.3 alone (data not shown). Thus based on the analysis described so far, the *p* <0.01 and RF ≥ 1.3 cutoff values were selected for general use when applying RATE for inference of HLA restrictions.

### Identification of promiscuous recognitions

In certain cases, the same epitope can be restricted by multiple alleles. These cases are denominated as promiscuous restrictions [16, 21], as opposed to the instances where a single HLA restricts the response (“monogamous restriction”). Promiscuous restrictions can be identified for an epitope by compiling multiple independent single allele determinations. Alternatively we had previously described an option for inferring promiscuous restrictions as part of RATE tool [8] using a combinatorial approach where the combined RF values are calculated for any combinations of alleles associated with positive RF values. However, when we examined this issue, we found that incorporation of combined combinatorial HLA restriction calculations as described in the earlier study [8] did not improve RATE performance (data not shown).

In the MTB data set, promiscuous restrictions were positively identified in 3 donors or more for six peptides, listed in Table 4. Because of this small number, the performance of the promiscuous option could not be fully evaluated in the present study. However, we examined whether these multiple restrictions could be identified by RATE as multiple independent associations for a given peptide. Indeed, we found that in approximately 50% of the cases the multiple restrictions were also independently inferred by RATE (those restrictions are in bold in Table 4). While this can be of help if promiscuous restrictions are of particular importance, we consider it more robust to go with the “monogamous restriction” calculations.

**Table 4:** Promiscuous restrictions in the MTB data set identified experimentally

Epitope	Promiscuous alleles
VDLAKSLRIAAKIYS	DQB1*06:02, DRB1*11:01, DRB3*02:02, DRB3*03:01
MSQIMYNYPAMLGHA	DQB1*06:02, DRB1*15:01
QAAVVRFQEAANKQK	DQB1*06:02, DRB3*02:02, DRB5*01:01
EISTNIRQAGVQYSR	DPB1*01:01, DPB1*04:01, DQB1*03:02, DQB1*06:02, DRB1*04:01, DRB1*04:04, DRB3*02:02, DRB4*01:01
MHVSFVMAYPEMLAA	DQB1*06:02, DRB5*01:01
ISTNIRQAGVQYSRA	DPB1*01:01, DQB1*03:02, DQB1*06:02, DRB1*04:01, DRB1*04:04, DRB3*02:02, DRB4*01:01, DRB5*01:01

The restrictions that were also inferred by the RATE tool are bolded

### Use of RATE to identify new restrictions and validation on a new data set

While experimental determinations of HLA restriction based on HLA matched APCs or single cell transfectants are by definition limited to those HLA molecules for which such reagents are available, the RATE method is not bound by this limitation. To illustrate this point we generated an output from RATE to highlight new HLA allele restrictions inferred from the MTB data described above, for which cell lines were not available to enable experimental determination. In total, 40 new restrictions were identified, demonstrating how the number and breadth of potential restrictions can be expanded by the use of RATE. The newly identified HLA restrictions are given in Table 5.

**Table 5:** New restrictions identified from MTB data for which cell lines were not available

Epitope	Allele	A^+^R^+^	Relative frequency	*p*-value
AAFSRMLSLFFRQHI	DPB1*03:01	2	13.667	0.005
AAVLRFQEAANKQKQ	DRB1*15:03	3	10.600	0.000
AAVVRFQEAANKQKQ	DRB1*15:03	6	3.600	0.001
AEKFKEDVINDFVSS	DQB1*03:03	2	13.500	0.005
AGWLAFFRDLVARGL	DRB4*01:03	5	2.692	0.010
AHGETVSAVAELIGD	DRB1*15:01	4	6.480	0.001
AHGETVSAVAELIGD	DRB5*01:01	4	3.600	0.008
ALSRVQSMFLGTGGS	DRB1*15:03	3	11.857	0.000
AQAAVVRFQEAANKQ	DRB1*15:03	7	4.375	0.000
ARTISEAGQAMASTE	DQB1*03:19	5	5.303	0.000
ATSLDTMTQMNQAFR	DQB1*06:01	2	27.000	0.001
ATSLDTMTQMNQAFR	DRB1*15:02	2	27.000	0.001
AYGSFVRTVSLPVGA	DQB1*02:02	5	3.661	0.003
AYGSFVRTVSLPVGA	DRB4*01:03	7	2.870	0.001
DLVRAYHSMSSTHEA	DPB1*04:02	3	5.640	0.006
HEANTMAMMARDTAE	DPB1*13:01	4	3.733	0.007
ILPIAEMSVVAMEFG	DQB1*03:03	3	10.125	0.001
ILPIAEMSVVAMEFG	DRB1*04:07	2	10.125	0.009
IQGNVTSIHSLLDEG	DRB4*01:03	7	2.344	0.007
LENDNQLLYNYPGAL	DRB1*15:02	3	6.231	0.003
LRIAAKIYSEADEAW	DQB1*03:19	6	2.893	0.003
MHVSFVMAYPEMLAA	DRB1*15:03	4	5.929	0.001
MLGHAGDMAGYAGTL	DPB1*13:01	2	9.333	0.010
MSQIMYNYPAMLGHA	DQB1*06:01	3	4.667	0.008
MSQIMYNYPAMMAHA	DQB1*06:01	4	3.682	0.004
MSQIMYNYPAMMAHA	DRB1*15:02	4	3.682	0.004
MTSRFMTDPHAMRDM	DQB1*06:01	2	13.500	0.004
MTSRFMTDPHAMRDM	DRB1*15:02	2	13.500	0.004
MVAAASPYVAWMSVT	DRB1*15:02	2	17.667	0.002
MVAAASPYVAWMSVT	DQB1*06:01	2	11.778	0.006
QAAVVRFQEAANKQK	DRB1*15:03	9	3.500	0.000
RQSGATIADVLAEKE	DRB5*01:01	5	3.214	0.005
RRMWASAQNISGAGW	DQB1*06:01	2	27.000	0.001
RRMWASAQNISGAGW	DRB1*15:02	2	27.000	0.001
VAAAQMWDSVASDLF	DQB1*06:01	2	11.778	0.006
VEDEARRMWASAQNI	DQB1*06:01	2	27.000	0.001
VEDEARRMWASAQNI	DRB1*15:02	2	27.000	0.001
VRFQEAANKQKQELD	DRB1*15:03	6	5.156	0.000
YNYPAMLGHAGDMAG	DRB1*15:02	2	9.333	0.010
YQAWQAQWNQAMEDL	DQB1*03:19	2	18.667	0.002

To further illustrate this point we also analyzed data from a study on human T cell reactivity to Timothy grass [17–19], where a set of 66 peptides was tested for reactivity in 137 allergic donors who expressed 99 unique alleles. In all, this represented a total of 6,534 possible peptide/HLA combinations. When focusing on HLA/peptide combination for which at least one donor was positive, the number of potential restrictions is reduced to 3,291. By applying the *p* <0.01 and RF ≥ 1.3 cutoff values, which was found to be optimal according to the analysis described above, we could further reduce the combinations to five restrictions, attributed to 3 unique peptides, (Tables 6 and 7), further exemplifying the usefulness of the method.

**Table 6:** Details of HLA restrictions from Timothy grass data set

Total number of peptides	66
Total donors tested	137
Total number of unique alleles expressed	99
Total HLA/peptide combinations	6,534
Total HLA/peptide combinations that gave positive response in at least one donor	3,291
Potential restrictions with *p* <0.05 and RF ≥ 1.25	5

**Table 7:** Newly identified restrictions from Timothy grass data set

Epitope	Allele	A^+^R^+^	RF	*p*-value
AVMLTFDNAGMWNVR	DPB1*01:01	5	3.063	0.006
ELRKTYNLLDAVSRH	DRB1*15:01	7	2.227	0.007
	DRB5*01:01	11	2.139	0.000
GEVLNALAYDVPIPG	DRB1*04:01	5	2.912	0.007
	DRB4*01:03	5	2.912	0.007

## Discussion

Here, we have utilized experimental data generated from an independent epitope identification study to validate and further optimize the performance of the RATE tool [8], developed earlier to infer HLA restrictions based on HLA typing and immune response data in human populations. Specifically, the present study takes advantage of a recently described data set, where HLA restrictions were experimentally determined for a set of 191 different MTB derived peptides tested in 63 MTB infected South African donors. We found that, on this data set, RATE was associated with a performance of MCC = 0.451 when optimal cutoff values for the output parameters were applied. Furthermore, the tool was associated with an accuracy of 0.745 and sensitivity of 0.550. In a practical sense, this performance indicates that the tool would allow a user to greatly reduce the number of potential restrictions to be examined, while still identifying about half of the true restrictions without any experimental work. The reason for the relatively low sensitivity is likely due to the fact that several restrictions occur infrequently and are thus not detected by an association based approach. In this respect, the fundamental utility of RATE from the viewpoint of an experimental user is that it identifies the most frequently occurring restrictions, thereby facilitating more efficient use of precious laboratory reagents and donor samples for subsequent analyses.

In utilizing the experimental data set to optimize tool performance, first consideration was given to the reliability of determinations as judged by the associated *p*-value in a Fisher’s exact test. Perhaps not surprisingly, we found optimal tool performance by considering only restrictions associated with a *p*-value <0.01. Interestingly, the performance of the tool is decreased by imposing significance levels less than 0.01. This has particular significance in terms of the potential use of Bonferroni correction, which we considered in the RATE tool output. The results clearly indicated that a Bonferroni correction should not be used, as it would not improve the tool performance, but rather essentially result in no useful inferences.

Imposing an additional requirement for an A^+^R^+^ threshold which result in selection of more reliable inferences, namely those HLA/epitope combinations based on epitopes recognized by multiple donors expressing a specific allele improved the performance compared to *p*-value cutoff alone. For this particular data set the MCC improved to 0.497 when A^+^R^+^ ≥ 5 cutoff was applied along with *p* <0.05. However, we do not recommend using this as a general threshold, because the optimal A^+^R^+^ threshold is expected to be strongly dependent on the absolute number of donors associated with a particular data-set. This type of filter could nevertheless be considered and adjusted to fit the experimental context, such as when a relatively large number of inferred restrictions can be feasibly tested, or whether it is desired to test only few higher probability candidates.

Following a different approach, we saw that increasing the magnitude of the associations, as measured by RF values improve RATE performance. In the present analysis we empirically determined and applied an optimal performance for RF value of 1.3. The RF threshold can be easily adjusted if more or less stringency is desired. We further emphasize how adjusting the RF value is indirectly correlated with *p* and A^+^R^+^ values. For this reason, in most cases adjusting the *p*-value threshold will also implicitly select for higher RF or A^+^R^+^ values as well.

In terms of further refinements, surprisingly we found that incorporating the predicted HLA binding in the restriction scheme was optimal when used in isolation for an IEDB consensus percentile rank of 15.0 but did not improve the performance in a broad range of percentile ranks (5.0 to 25.0) when used in combination with other optimized parameters. Several different factors might contribute to this result. First, it is well established that HLA binding is a necessary but not sufficient requirement for T cell recognition; in the case of HLA restrictions, all peptides studied are by definition binders to some of the alleles, and the well-known promiscuity observed in the case of HLA class II binding [22] might hinder realizing any increase in RATE performance based on HLA binding predictions. Second, it is possible that the result reflects that HLA class II binding predictions for certain alleles may be relatively inaccurate. This concern will be addressed in future by progressive retraining of HLA class II prediction tools, as more HLA binding data becomes available, and increased accuracy can be achieved.

We also found that the iterative combination of different allele subsets described in the previous study [8] did not improve RATE performance. However, in the MTB data set that was used to optimize the performance only 6 out of the total 191 peptides had promiscuous restrictions and for this reason this data set was not ideal to address the best strategy to identify promiscuous restrictions. Future studies utilizing a larger number of experimentally determined promiscuous restrictions will be required to fully evaluate this issue. At the same time it should be considered that loss of significance due to multiple comparisons is a serious problem for the promiscuous option. To truly demonstrate promiscuous restrictions might require larger data sets than the one utilized here (which is representative of most epitope identification studies). Based upon these considerations it is recommended that the “monogamous restriction” calculation be used for practical purposes (monogamous refers to HLA-peptide relationship where a peptide is found to be restricted by a single allele). To demonstrate or identify potential promiscuous restrictions it seems safer to record different HLA restrictions independently identified for a given epitope.

Finally the RATE tool was applied to infer additional restrictions both in the original data set, and in a data set including epitopes derived from pollen allergens. In the present study, we adjusted the RATE parameters according to a known MTB data set. In future studies it will be interesting to assess the performance of RATE on unknown samples to exclude overfitting of the parameters to the MTB data. However, these experiments are laborious and expensive and therefore beyond the scope of the current study.

The results highlight how the RATE approach is suited for inference of restrictions for which no transfected cell lines are available. We emphasize that these instances most often correspond to alleles that are rare in the general population, but relatively frequent in a specific study population, ethnicity or geographical location. In this respect, it is notable that several of the new restrictions inferred by the RATE tool in the MTB data set were mediated by the HLA DRB1*15:03 allele, which is present at 0.0517 frequency in the Western Cape region study population, 0.0596 frequency in South Africa, but only at 0.0225 worldwide (http://www.allelefrequencies.net [23]). These results emphasize the value of the RATE tool in terms of providing HLA restriction data in the context of diverse HLAs and complex multi-ethnic human trials.

## Additional files

Additional file 1: RATE_validation_Additional_file_1.xlsx: Input data used for determining HLA restrictions using RATE. (XLSX 75 kb)

Additional file 2: RATE_validation_Additional_file_2.xlsx: Analysis report generated by RATE for each peptide/allele combinations in the MTB data set used for identification of HLA allele restriction by RATE. (XLSX 1370 kb)

Additional file 3: RATE_validation_Additional_file_3.xlsx: Positive and negative restrictions of the 102 HLA/peptide combinations derived from the HLA restrictions determined experimentally using transfected cell lines. (XLSX 12 kb)

Additional file 4: RATE_validation_Additional_file_4.xlsx: Effect of different cutoff values for A^+^R^+^ on RATE results. (XLSX 10 kb)

Additional file 5; RATE_validation_Additional_file_5.xlsx: Effect of different predicted binding (IEDB consensus percentile rank) cutoffs on RATE results. (XLSX 9 kb)

## Abbreviations

HLA: Human leukocyte antigen
IGRA: Interferon-gamma release assay.
MCC: Matthews correlation coefficient
MHC: Major histocompatibility complex
MTB: *Mycobacterium tuberculosis*
PBMCs: Peripheral blood mononuclear cells
RAST: Radioallergosorbent test
RATE: Restrictor analysis tool for epitopes
RF: Relative frequency
SFC: Spot-forming cell
TG: Timothy grass
